# Navigating the Difficult Robotic Assisted Pyeloplasty

**DOI:** 10.5402/2012/291235

**Published:** 2012-10-30

**Authors:** David D. Thiel

**Affiliations:** Department of Urology, Mayo Clinic, 3 East Urology, 4500 San Pablo Road, Jacksonville, FL 32224, USA

## Abstract

Pyeloplasty is the gold standard therapy for ureteropelvic junction obstruction. Robotic assisted pyeloplasty has been widely adopted by urologists with and without prior laparoscopic pyeloplasty experience. However, difficult situations encountered during robotic assisted pyeloplasty can significantly add to the difficulty of the operation. This paper provides tips for patient positioning, port placement, robot docking, and intraoperative dissection and repair in patients with the difficult situations of obesity, large floppy liver, difficult to reflect colon (transmesenteric pyeloplasty), crossing vessels, large calculi, and previous attempts at ureteropelvic junction repair. Techniques presented in this paper may aid in the successful completion of robotic assisted pyeloplasty in the face of the difficult situations noted above.

## 1. Introduction

Pyeloplasty is the gold standard therapy for ureteropelvic junction obstruction (UPJO), with reported success rates approaching 90% [1]. Urology has embraced the da Vinci surgical system (Intuitive Surgical, Inc., Sunnyvale, CA, USA) for complex reconstructions of the urinary tract, including pyeloplasty. Success rates of robotic assisted pyeloplasty (RAP) appear to be equivalent to open pyeloplasty while conferring the well-published advantages of minimally invasive surgery (decreased postoperative pain, shorter hospital stay, quicker return to normal activities, etc.) [1–3]. Situations encountered during RAP can significantly alter surgical difficulty and possibly contribute to surgical morbidity. Obesity, large floppy liver, unretractable colon, crossing vessels, large calculi, and previous attempts at UPJO repair can all present a significant intraoperative difficulty. This paper describes techniques to aid in the successful completion of RAP if these situations are encountered.

## 2. Standard Technique

**Figure 1:**
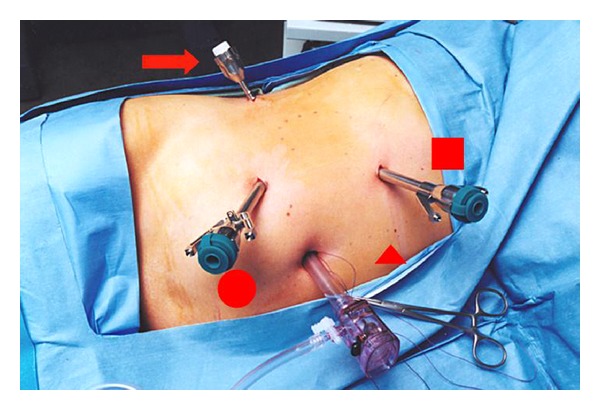
Standard port placement for RAP. 8 mm robotic ports are placed in the upper and lower quadrant midclavicular lines. The 12 mm camera port is placed near the umbilicus. A 12 mm assistant port is placed in the suprapubic midline (circle). In right-sided cases, a midaxillary 5 mm port (arrow) may be needed for liver retraction. In obese patients, the assistant port may need to be moved to the subxiphoid region (square) or the midline (triangle) (assuming that the robotic ports have been moved laterally). (Figure adapted from [5] with permissions from the puplisher).

The standard patient positioning, port placement, colon mobilization, UPJO dissection, repair, and stent placement as well as postoperative management of RAP have been well described [4]. The patient is placed in a 70-degree flank position with the ipsilateral arm secured above the head on an arm board. A cystoscopically placed stent-wire complex is anchored to a urethral catheter and prepared in the sterile operative field. Standard port placement is demonstrated in Figure 1. The colon and its mesentery are reflected medially to reveal the underlying kidney, renal pelvis, and ureter. The renal pelvis and proximal ureter are freed of their surrounding attachments with care taken to avoid excessive manipulation of the ureter and UPJ in order to preserve peri-ureteral blood supply. It should be noted that we complete our dissection with three robotic arms. We have not found the fourth robotic arm helpful or necessary in pyeloplasty surgery.

The stenotic UPJ segment is excised and sent to pathology as a permanent specimen. The ureter is spatulated anteriorly as viewed (since the patient is in flank position, this is an anatomically lateral spatulation) for the length of the robotic scissors. 3-0 Vicryl sutures on an RB-1 needle are used to reconstruct the UPJ in an interrupted fashion and excess renal pelvis is closed as needed. Prior to completing the anastomosis, the open-ended ureteral catheter is exchanged for a 7 French double-J stent of the appropriate length. This is done by the table-side assistant over the previously placed wire. The robotic surgeon can hold the wire in the renal pelvis to provide two-point traction and prevent wire kinking in the bladder [4]. The urinary catheter is removed on postoperative day 1 and patients are placed on a clinical pathway to be discharged home on day 2. The ureter stent is removed 3-4 weeks following surgery.

## 3. Robot Docking

**Figure 2:**
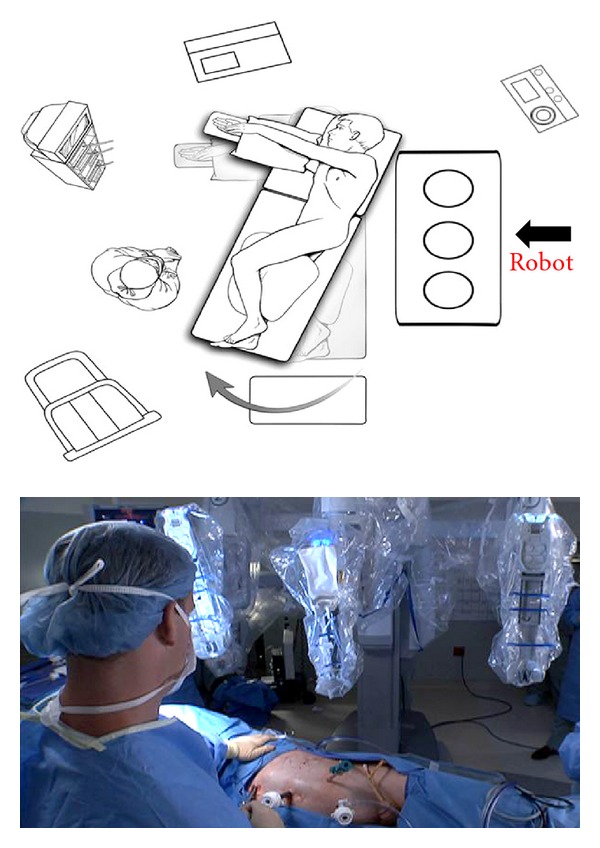
Robot docking for RAP. The patient's legs are shifted toward the operating surgeon, and the robot is brought in perpendicular to the room. This creates a 60 degree angle with the patient's spine and helps instrument mobility in the upper quadrants. (Figure adapted from [5] with permissions from the publisher.)

A full range of motion of the robotic instruments is imperative when difficult RAP situations are encountered. Proper da Vinci robot docking can be one of the more difficult aspects of the operation. The robot should be brought in from the patient's posterior side. The initial impulse is to bring the robot in at a 90-degree angle to the patient, but this placement will limit instrument use in the upper quadrants [4]. We find it helpful to dock the robot at a 60-degree cephalad angle with respect to the patient's spine. The patient's legs are shifted toward the operating surgeon, and the robot is brought in perpendicular to the room and not the operating table (Figure 2).

## 4. Difficult Situations

### 4.1. Obesity

Obese patients or those with large body habitus can pose technical challenges in a variety of surgical procedures. Obese patients may have a greater number of medical comorbidities, which may increase the risk of perioperative complications. The patients should have a thorough medical evaluation before proceeding with surgery. The authors have noted an increased risk of Clavien grade >3 complications in obese men (BMI > 30 kg/m^2^) undergoing robot assisted laparoscopic prostatectomy [6]. 

Ports may need to be shifted laterally in obese patients, although the extended length instruments available with the da Vinci S or Si systems may be more forgiving with regards to skin-to-target distance [6]. The largest difficulty we have found in obese patients is the location of the assistant 12 mm port. This port is used for retraction, suction, suture passing, and suture cutting. The standard location of the suprapubic midline is often too far away from the operative target in obese individuals. In obese patients we place the assistant port in the subxiphoid region or the midline (assuming that the ports have been shifted laterally) (Figure 1). The surgeon should insure that the assistant port can reach the operative target without hitting one of the working ports. The port should not be “stacked” directly behind one of the robot ports or the camera port.

### 4.2. Large Floppy Liver

In right-sided pyeloplasties, a large floppy liver can be burdensome. If that is the case, we place a midaxillary 5 mm port on the right side (Figure 1) and utilize a table anchored Snowden-Pencer liver retractor to nudge the liver in a superior-medial location. There has been some concern from Intuitive Corporation personnel about posterior table-mounted instrumentation such as this, but we have not found robot docking to be a problem with the standard system or the Si system utilizing this equipment.

### 4.3. Redundant Colon

The standard intraperitoneal pyeloplasty involves colon reflection to expose the ureter and kidney. In certain instances, the colon (especially the left colon in thin patients) may be very difficult to reflect. The colon may be large, redundant, and draped over an extremely large UPJO. Often times in these patients, the colon mesentery lies directly over the UPJO. Left-side laparoscopic transmesenteric kidney surgery has been successfully performed [7, 8]. A longitudinal hole is made in the mesentery followed by blunt and sharp dissection to isolate the UPJO in the standard fashion. If transmesenteric pyeloplasty is to be used, it is imperative to avoid the mesenteric vasculature during dissection. 

Advocates of this technique note that their operating time is quicker for left-side laparoscopic pyeloplasty, and the lack of colon mobilization may decrease the chances of postoperative ileus [7]. There is a risk of injury to the colon mesentery utilizing this procedure but there are no reported cases of bowel necrosis utilizing this technique in the literature. Others have hypothesized that an intact marginal arcade should avoid vascular compromise of the colon in the case of inadvertent mesenteric vascular injury [7, 8]. Romero et al. noted that this technique can be used easily in thin patients. Although obesity is not a contraindication to this approach, heavier patients tend to have thicker mesentery, which may make transmesenteric dissection much more difficult [7]. Reports utilizing this technique note reapproximation of the mesentery with 4-zero suture following the procedure to prevent the theoretical risk of herniation [7]. We have found this difficult and no longer perform mesenteric closure. To date we have not had bowel herniation following the transmesenteric approach.

### 4.4. Crossing Vessels

Crossing vessels are present in 38–71% of UPJO cases and 20% of normal kidneys [9, 10]. If unrecognized during UPJO dissection, these crossing vessels can cause troublesome bleeding. Care should be taken to preserve these vessels during dissection. The vessels are dissected with a blunt dissector in the left hand and monopolar scissors in the right hand. Circumferential dissection of the vessels is completed until they are completely free of the underlying tissue. Once the UPJ is transected, repair usually proceeds anterior to the crossing vessels. Boylu et al. have questioned the necessity of transposing all UPJ repairs anterior to the crossing vessels and have had success leaving the UPJ posterior to the crossing vessels if the anastomosis will be less than 1 cm from the vessels [9]. This data confirms that it may be safe to leave the vessels posterior to the UPJ repair if it appears anatomically correct intraoperatively.

### 4.5. Large Calculi

For patients with large stones or renal pelvis stones of any size with simultaneous UPJO, RAP provides an excellent opportunity for repair of UPJO and stone removal. Simultaneous laparoscopic UPJO repair and stone extraction have been well described, and there are currently 39 reports of concomitant RAP and pyelolithotomy in the literature [11]. Large calculi can induce inflammation that renders tissue edematous and friable making UPJO repair cumbersome and difficult [12]. Port placement and instrument utilization should not be altered from standard RAP. Occasionally, the robot can limit large movements in a fixed space that are better performed with laparoscopic instruments. In this particular case the procedure was completed robotically once the stone was extracted.

We prefer to place the stone in a collection bag and remove it following the UPJ reconstruction. If calyceal stones or smaller residual fragments are present at the time of UPJ repair, they can be accessed via cystoscope inserted through an abdominal port. We find that renal access is maximized if the scope is inserted through the most cephalad port. The stones can then be grasped with a basket or fragmented with the holmium laser. A review of robotic stone extractions by Badalato et al. noted that staghorn calculi had a much higher rate of open conversion, secondary procedures, and residual stone burden [11]. 

### 4.6. Revision Surgery

Perhaps the most challenging situation encountered during RAP is that of failed previous attempts at UPJO repair. Previous pyeloplasty, endopyelotomy, or balloon dilation can all cause inflammatory tissue and fibrosis that make UPJ dissection extremely difficult. Endopyelotomy is the current first-line therapy for failed UPJO repairs with acceptable success rates [13, 14]. Success rates of 84–89% have been noted with laparoscopic UPJO repair following previous failed therapies [15, 16]. The authors of these studies note the longer operative time and difficult dissection associated with revision UPJO surgery. Others have proposed that robotic assistance offers better visualization and delineation of tissue planes when severe scarring is present. This allows for dissection to proceed while preserving peri-ureteral blood supply [17]. It remains to be seen whether this allowance translates into higher success rates.

For revision pyeloplasty, we believe it is imperative to have a ureteral localization stent in place to aid in ureter identification intraoperatively. Caution should be utilized when dissecting around the region of the UPJ as missed lower pole vessels (from previous pyeloplasty) have been noted in 22.2% of revision RAP surgery [17]. The same instruments and dissecting techniques as standard pyeloplasty are utilized. If scarring is prohibitive, the dilated renal pelvis can be incised early. This provides an excellent gripping point for retraction and the inflamed tissue can be swept off of the remaining portions of the UPJ. All pyeloplasties are completed in a dismembered fashion at our institution, although flap procedures have been discussed as helpful in revision pyeloplasty [15, 16]. Patients undergoing revision pyeloplasty at our institution receive the same postoperative care and planning as patients undergoing standard pyeloplasty.

## 5. Conclusion

The learning curve for intracorporeal suturing is much shorter with the da Vinci surgical system than with conventional laparoscopic instruments [18]. Robotic technology has allowed surgeons without laparoscopic pyeloplasty experience to successfully perform robotic assisted laparoscopic pyeloplasty [19]. With the increasing popularity of the da Vinci robot, it seems that laparoscopic pyeloplasty will become available to a broader range of urologists without laparoscopic experience. Techniques described in this paper may aid in successful completion of RAP in the face of the difficult intraoperative situations described.
